# Short echo time dual-frequency MR Elastography with Optimal Control RF pulses

**DOI:** 10.1038/s41598-022-05262-3

**Published:** 2022-01-26

**Authors:** Pilar Sango-Solanas, Kevin Tse Ve Koon, Eric Van Reeth, Helene Ratiney, Fabien Millioz, Cyrielle Caussy, Olivier Beuf

**Affiliations:** 1 Univ Lyon, INSA Lyon, Inserm, UCBL, CNRS, CREATIS, UMR5220, U1294, 69621 Villeurbanne, France; 2 CPE Lyon, Département Sciences du Numérique, Lyon, France; 3 Univ Lyon, INSERM, INRA, INSA Lyon, UCBL, CarMen Laboratory, 69495 Pierre-Bénite, France; 4 Hospices Civils de Lyon, Hôpital Lyon Sud, Département Endocrinologie, Diabète Et Nutrition, 69495 Pierre-Bénite, France

**Keywords:** Biomedical engineering, Diagnostic markers

## Abstract

Magnetic Resonance Elastography (MRE) quantifies the mechanical properties of tissues, typically applying motion encoding gradients (MEG). Multifrequency results allow better characterizations of tissues using data usually acquired through sequential monofrequency experiments. High frequencies are difficult to reach due to slew rate limitations and low frequencies induce long TEs, yielding magnitude images with low SNR. We propose a novel strategy to perform simultaneous multifrequency MRE in the absence of MEGs: using RF pulses designed via the Optimal Control (OC) theory. Such pulses control the spatial distribution of the MRI magnetization phase so that the resulting transverse magnetization reproduces the phase pattern of an MRE acquisition. The pulse is applied with a constant gradient during the multifrequency mechanical excitation to simultaneously achieve slice selection and motion encoding. The phase offset sampling strategy can be adapted according to the excitation frequencies to reduce the acquisition time. Phantom experiments were run to compare the classical monofrequency MRE to the OC based dual-frequency MRE method and showed excellent agreement between the reconstructed shear storage modulus G′. Our method could be applied to simultaneously acquire low and high frequency components, which are difficult to encode with the classical MEG MRE strategy.

## Introduction

Magnetic Resonance Elastography (MRE) is a valuable non-invasive technique for the quantification of mechanical properties of tissues based on the measurement of the characteristics of shear waves propagating through the organ of interest^1^. Many diseases alter the morphology and structure of the affected organs, producing measurable modifications of the mechanical properties of tissues. These alterations can be quantified noninvasively with certain imaging methods^2^ allowing the staging and longitudinal follow up to detect the progression or regression of a disease. For example, in the case of liver fibrosis, MRE is a validated clinical tool since it provides results comparable to liver biopsy^3–6^ while being non-invasive and enabling the assessment of the whole liver contrary to liver biopsy which probes only a small liver sample. Indeed, MRE is now a recognized modality for the assessment of liver fibrosis stage in patients with nonalcoholic fatty liver disease (NAFLD)^5,7^. Many other organs targeted in clinical applications such as the heart^8–10^, brain^11–14^ and breast^15–18^ benefit also from the use of MRE as a tissue mechanical characterization technique.

Initially, Lewa et al.^19^ proposed the use of mechanical waves with MRI to image the elastic properties of materials. Their method was based on the detection of Larmor frequency modulations caused by the application of mechanical waves^20^. Afterwards, Muthupillai et al.^1^ proposed the application of oscillating motion encoding gradients (MEG) synchronized to the mechanical excitation to encode nuclear spins motion into the phase of the NMR signal. This technique has been largely exploited over the last decades and has become the classical strategy to carry out MRE.

Usually, the MEG is placed after the excitation and before signal acquisition and it oscillates at the same frequency as the mechanical excitation. Therefore, the minimum echo time TE is fixed by the duration of the MEG, resulting in a degradation of the signal to noise ratio (SNR), especially when low excitation frequencies are used on tissues having short T2 values. This effect is less important when using high excitation frequencies since the excitation periods are shorter. However, high frequencies are rapidly attenuated and the gradient system of most MRI scanners cannot reach high frequencies due to slew rate limitations.

The presence of oscillating MEGs complicates the use of the classical technique for some applications such as cardiac MRE, which needs short TE, low vibration frequencies and ECG triggering. This is the reason why some novel motion encoding strategies have been studied. First, the fractional encoding method introduced by Rump et al.^21^ allowed to overcome the drawback of the SNR degradation at low excitation frequencies since they proposed to use high frequency MEGs to encode them. The duration of MEG is thus shorter than the excitation wave period. This allowed the encoding of low frequencies with shorter TEs at the expense of a reduced encoding efficiency. Then, encoding strategies that do not use oscillating gradients were also proposed. For instance, a displacement encoding with stimulated echoes (DENSE) Elastography sequence^22,23^ was proposed to encode low frequency motion in tissues exhibiting short T2 values. Short encoding gradients that are independent of the excitation wave period are used, enabling short TEs. However, the DENSE MRE strategy presents a loss of signal amplitude compared to the classical method.

Instead of using oscillating gradients to encode the oscillatory motion, the usage of radiofrequency (RF) field gradients was also proposed to detect motion from spectroscopic data by relating the NMR signal as a function of the wave frequency excitation^24,25^. This method was then validated to acquire 2D magnitude images showing shear waves propagating at frequencies in the kilohertz range in phantom experiments^26^. However, this method was limited in terms of RF power since a very high power was needed to obtain the desired B1 gradient.

More recently, the application of optimized RF pulses, designed with an Optimal Control (OC) algorithm, in presence of a constant gradient was investigated^27,28^. OC theory was demonstrated to be useful in MRI in the context of image contrast optimization^29,30^ and robust excitation and refocusing^31–33^. Both mentioned applications are based on the NMR signal magnitude whereas in the case of MRE, OC pulses were proposed for phase contrast applications such as MRE^28^. The ability of such pulses to control the magnetization phase was first validated^27,34^. Then, it was demonstrated that the simultaneous application of an OC based pulse and a constant gradient allows the encoding of an oscillatory motion^28^. The pulse brings the magnetization from the equilibrium state to the desired target state that is related to the phase of the wave propagation. The OC pulse, applied with a constant gradient during the mechanical excitation, simultaneously performs spatially selective excitation and motion encoding in slice direction. As no oscillating gradients are required after the excitation, this approach enables short TE and avoids fast gradient switches, which facilitates the application of low and high frequency MRE. However, the constant gradient imposes that the motion encoding direction be similar to the slice selection direction. For now, this strategy has been applied to carry out single-frequency MRE with the above conditions^35^.

Currently, multifrequency MRE is increasingly being explored because the parameters commonly used to quantify the viscoelastic behavior of biological tissues are frequency dependent. The dispersion of the real and imaginary components of the complex shear modulus (G*) can provide information about the microstructure of tissues^36–39^. The alterations of the viscoelastic properties of tissues at a microscopic scale caused by some diseases can be detected with the dispersion results fitted to a powerlaw^40–42^.

Multifrequency results can be acquired separately with monochromatic wave excitations at multiple frequencies or simultaneously. The simultaneous encoding of multiple frequencies was investigated by some authors. The fractional encoding method was extended to multifrequency acquisitions. It was successfully used on human liver^40,43,44^ to simultaneously encode four sinusoidal waveforms with frequencies of 25.0, 37.5, 50.0 and 62.5 Hz using a 50 Hz sinusoidal MEG. Since then, the concept of fractional encoding has been exploited in different studies to further its benefits^45,46^. For example, Garteiser et al.^45^ proposed a MRE sequence to acquire multiple frequency components on several slices in a single examination. However, the fractional encoding method is limited to low frequency vibrations; it is thus not suitable for small samples where high frequencies are needed.

On the other hand, a recent study proposed a harmonic wideband encoding MRE method^47^ using the ability of low frequency trapezoidal MEGs to encode higher frequencies, thus overcoming gradient limitations of the MR scanner. The harmonic wideband method allowed the encoding of odd multiples of the fundamental frequency. It was thus shown on phantom experiments that it could simultaneously encode 300 and 900 Hz using a 300 Hz trapezoidal MEG^47^. Nevertheless, as the harmonic wideband encoding method is more appropriate for high frequencies, it could not be easily applied to the typical targeted organs in MRE such as the liver since the high frequency shear wave component would be rapidly attenuated.

These simultaneous multifrequency MRE strategies allow the reduction of the total examination time compared to the classical method encoding where only one frequency is encoded per acquisition. Despite this, the limitation imposed by the presence of the oscillating gradients remains. Hence, being able to simultaneously encode several vibrations covering a large range of frequencies without the application of MEG while reducing the examination duration could be an important added value to MRE methods.

In response to this proposal, we redesigned the OC algorithm applied to MRE in order to extend the motion encoding strategy to more than one frequency component. Therefore, the aim of this study is to validate the use of OC RF pulses to simultaneously encode a dual-frequency shear wave at short TE. Phantom experiments were carried out to validate the applicability of the proposed approach on a preclinical 7T MRI system. A good agreement of the shear storage modulus values was found when compared to classical monofrequency MRE experiments.

## Optimal Control theory

### Optimal Control algorithm

Through the application of the Pontryagin Maximum Principle (PMP)^48^, Optimal Control theory enables the computation of the control parameter and the associated optimal trajectory of a dynamic system with respect to a defined target state and its associated cost function. The numerical approach used to solve this optimal control problem is the GRAPE (GRadient Ascent Pulse Engineering) algorithm which is a gradient ascent algorithm introduced for Nuclear Magnetic Resonance pulse design^49^. This algorithm updates the control parameter and the trajectories that satisfy the optimal conditions to minimize the cost function at each iteration, while satisfying the constraints imposed by the PMP.

The dynamic system in the case of MRI corresponds to the macroscopic magnetization $\vec{M}$ of isochromats, whose evolution can be modelled by Bloch equations. The control parameter corresponds to the components of the RF pulse ($\vec{U} = (u_{x} ,u_{y} )$), which will manipulate the macroscopic magnetization from a given steady state to the desired target state.

The target states are defined by the user depending on the application. For instance, they can be defined so as to optimize the magnitude of the macroscopic magnetization while ensuring the robustness of the pulse on a B_0_ inhomogeneity range^29^. Likewise, they can be defined as phase patterns so as to manipulate the phase of the macroscopic magnetization in the case of phase contrast mechanisms such as MRE^27^.

### Optimal Control applied to multifrequency MRE

**Figure 1:**
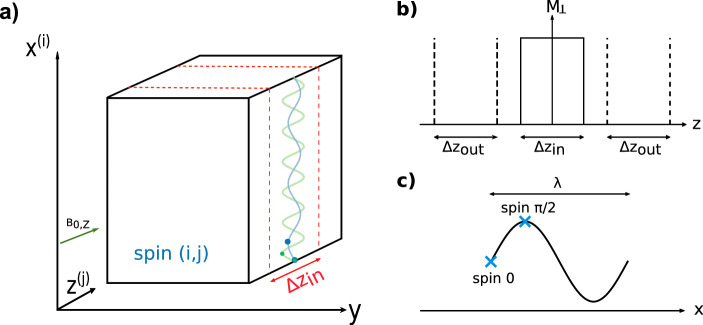
(**a**) Experimental setup: the shear wave propagates in the phantom along the x-axis. The motion sensitizing direction is fixed to the slice gradient direction. (**b**) An axial slice is acquired with a corresponding bandwidth Δz_in_ and controlled outbands Δz_out_ along the z-axis, all of which are discretized. (**c**) Isochromats are discretized along the shear wave propagation direction (x-axis) and are defined by θ_i_ = 2πx_i_/λ with x_i_ representing a specific position.

To perform MRE with OC, the RF pulse is simultaneously applied with a constant gradient G_z_ to perform slice selection through the frequency selectivity of the RF pulse as it is classically done. Figure 1a presents the setup and coordinate system considered for the definition of the multifrequency MRE optimal control problem. In the optimization problem, we discretize J isochromats along the slice direction (taken to be z here). Each one is located at a position $z_{\left( j \right)}$. This implies the concept of bandwidth of the optimized RF pulse that is related to the desired slice thickness (∆z_in_). Two outbands (∆z_out_) are also considered (Fig. 1b) with a different target state. The RF pulse and a constant gradient are simultaneously applied during the propagation of the shear wave through the phantom, which induces a time varying B_0_ field for all considered isochromats. The encoding motion direction is thus imposed by the gradient direction and must be parallel to it. Isochromats are then discretized along an arbitrary wavelength direction $x_{\left( i \right)}$ (taken to be x here). Each one is described in terms of phase by $\theta_{\left( i \right)} = 2\pi \frac{{x_{\left( i \right)} { }}}{\lambda }$, which is independent to the propagation direction. In a previous study, it was demonstrated that considering only two isochromats separated by a quarter of a wavelength ($\theta_{\left( 1 \right)} = 0$ and $\theta_{\left( 2 \right)} = \pi /2$), it is possible to converge towards a pulse that continuously encodes the wave propagation in the magnetization phase while also reducing the computation time^27^. In the present study, only these 2 isochromats are considered (Fig. 1c). In the case of multifrequency MRE, a shear wave composed of K different frequency components ($f_{k}$) propagates through the phantom. Therefore, in order to adapt the algorithm to multifrequency MRE, we discretized also isochromats according to their excitation frequency. For this, we considered isochromats at K different frequencies. This made the optimization process more complex since the number of controlled isochromats was K times greater and there was no guarantee that such a pulse could be found.

Taking all this into account, the field variation perceived by isochromats located at (i,j) is given by:

1 $$\Delta B_{{0_{{\left( {i,j} \right)}} }} \left( t \right) = G_{z} \left( {\mathop \sum \limits_{k = 1}^{K} [A_{k} sin(2\pi f_{k} t + \theta_{\left( i \right)} )] + z_{\left( j \right)} } \right)$$

where $A_{k}$ represents the amplitude of each component of the shear wave. This expression of the resonance offset is included in Bloch equations for the optimization process.

Different target states are attributed whether the isochromat is located inside (∆f_in_ corresponding to ∆z_in_) or outside (∆f_out_ corresponding to ∆z_out_) the bandwidth. The target state for isochromats outside corresponds to the equilibrium state so as to have a proper slice selection. For the isochromats inside, the target state maximizes the transverse magnetization with a magnetization phase which is directly linked to the position of the isochromat along the wavelength of the oscillatory motion they are undergoing. The target states are thus defined by the following coordinate vectors:

2 $$\begin{array}{*{20}l} {\vec{T}_{{\left( {i,j} \right)}} = \left( {\rho \cos \theta_{\left( i \right)} ,\rho {\text{sin}}\theta_{\left( i \right)} , 0} \right)} \hfill & {if\,\,j \in \Delta f_{in} } \hfill \\ {\vec{T}_{{\left( {i,j} \right)}} = \left( {0,0,1} \right)} \hfill & {if\,\,j \in \Delta f_{out} } \hfill \\ \end{array}$$

where $\rho$ represents the magnetization amplitude varying between 0 and 1.

The cost function is defined in order to be minimized during the optimization process. It corresponds to the quadratic difference between the final magnetization states after the application of the RF pulse $\vec{M}_{{\left( {i,j} \right)}} \left( {t_{f} } \right)$ and the defined target states $\vec{T}_{{\left( {i,j} \right)}}$:

3 $$C\left( {\vec{U}} \right) = \mathop \sum \limits_{j = 1}^{J} \mathop \sum \limits_{i = 1}^{2} \mathop \sum \limits_{k = 1}^{K}\mid\mid \vec{M}_{{\left( {i,j} \right)}} \left( {t_{f} } \right) - \vec{T}_{{\left( {i,j} \right)}}\mid\mid^{2}$$

## Materials and methods

### Optimal Control RF pulse generation

Several RF pulses were separately optimized for different pairs of frequencies: (A) 300 and 900 Hz with respective wave amplitudes of 15 and 45 μm, (B) 300 and 400 Hz with respective wave amplitudes of 15 and 20 μm (C) 300 and 600 Hz with respective wave amplitudes of 15 and 30 μm and (D) 400 and 600 Hz with respective wave amplitudes of 15 and 30 μm. The amplitude of the high frequency component was increased with respect to the lower one in order to account for the greater attenuation of high frequencies and the reduction of the encoding efficiency of the MEG at high frequencies with the classical method. The pulse durations were fixed to 15 ms. As the relaxation times have to be taken into account during the optimization process, a prior knowledge of the T2 is needed. The pulse were optimized for a T1 value of 400 ms and a T2-value of 30 ms of our phantom. The pulse amplitudes were bounded during the optimization to 94 μT in order to respect the constraints of the coil and the RF amplifier used. Only the x-component of the RF pulse was optimized ($u_{x}$) thus further reducing the computation time. The slice bandwidths $\Delta f_{in}$ were set to 7 kHz, which corresponds to a 1 mm slice with a gradient amplitude G_z_ = 164 mT/m. The outer bandwidth intervals $\Delta f_{out}$ were set to $\pm$ [4.5, 10.5] kHz yielding a transition band of ± [3.5, 4.5] kHz. The frequency step was set to 10 Hz and 20 Hz in the slice and the outer bandwidth, respectively. Only two isochromats groups per wavelength (at $\theta_{\left( 1 \right)} = 0$ and $\theta_{\left( 2 \right)} = \pi /2$) were considered for each frequency component. This yielded a total of 4004 different isochromats which were simulated and controlled for every RF pulse. The total duration of the optimization process for one pulse was around 137 h on a computing grid of 4-core, 2.5 GHz machines.

### Robustness to the T2 variability

Biological tissues are often heterogeneous and have variable transverse relaxation times. As OC pulses are optimized for a given optimization T2 value, it is expensive in terms of computation time to generate several pulses for each specific case. The robustness of OC pulses to the variability of T2 can be evaluated by simulation. For this purpose, the OC RF pulse A was applied via the Bloch equations to several numerical phantoms with different T2 values. From these results, the simulated encoded phase $\varphi_{sim}$ defined here as the difference between the mean phase of each isochromat group ($\varphi_{sim} = \overline{\varphi }_{{sim_{{iso\,{\uppi }/2}} }} - \overline{\varphi }_{{sim_{iso\,0} }}$) and the simulated transverse magnetization were computed. To simulate the effect of the OC pulse when there is no mechanical excitation the pulse was also applied to the same numerical phantoms with a null motion amplitude. The simulated phase noise $\sigma_{sim}$ was then defined as the standard deviation of the isochromats phase in the absence of motion. Finally, similarly to the experimental Phase-to-Noise ratio (PNR), the simulated PNR_sim_ was calculated as the quotient of the simulated encoded phase divided by the phase noise ($\varphi_{sim} /\sigma_{sim}$).

### MRE setup

Acquisitions were performed on a preclinical 7 T MRI scanner (BioSpec Bruker system, Ettlingen, Germany), with a quadrature 72 mm inner diameter volume coil in transmit/receive mode.

Experiments were carried out on a uniform phantom composed of 75%-standard plastisol (Plastileurre Standard, Bricoleurre, France). Relaxation times measured using a RARE (Rapid Acquisition with Refocused Echoes) sequence with different TEs and TRs gave [T1, T2] = [420, 35] ms. Previous studies confirmed that the complex modulus of plastisol phantoms can be reliably assessed using MRE^50,51^.

A piezoelectric actuator (CEDRAT Technologies) was used as mechanical transducer driver. A waveform generator (Agilent 33220A) was configured to produce the sum of two harmonic signals with the above-mentioned frequencies and amplitudes.

### MRE acquisition

**Figure 2:**
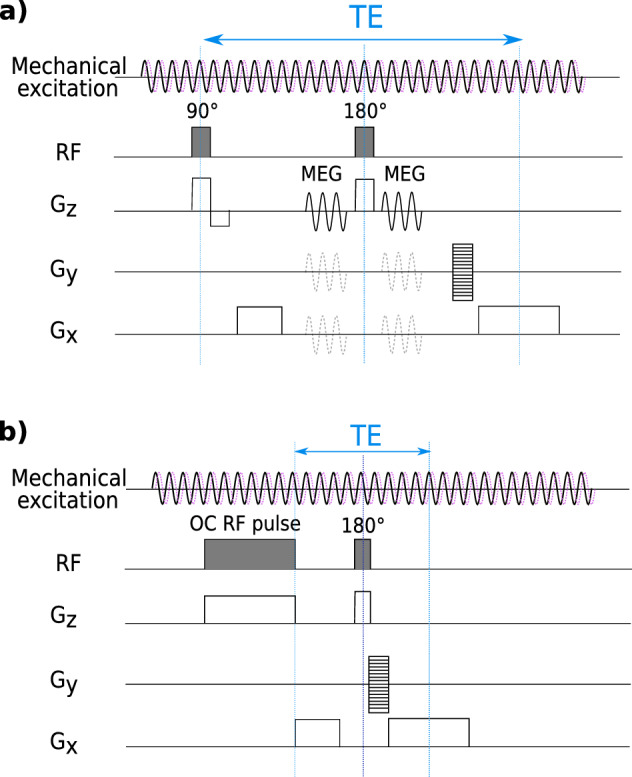
Sequence diagrams of (**a**) the classical MEG MRE and (**b**) the OC-based MRE used in this study.

As the objective of this study was to compare the classical monofrequency MEG method versus the OC-based multifrequency MRE proposed here, both type of acquisitions were carried out. The used sequences were based on a conventional turbo spin-echo sequence. For the classical method, one cycle of MEG with 164 mT/m maximum amplitude was added before and after the refocusing pulse. MEG was synchronized with the mechanical excitation. For the OC method, the calculated pulses were used as excitation pulse. The OC pulse itself was synchronized with the mechanical excitation. Since the OC approach actually requires the encoding of motion parallel to the slice direction, a single direction was sensitized. Both sequences are presented in Fig. 2.

Fixed acquisition parameters were axial slice orientation, TR = 1500 ms, RARE factor = 4, single slice of 1 mm thickness, field of view = 4 × 4 cm^2^, matrix size = 128 × 128.

To avoid artifacts during the inversion process for the viscoelastic parameters computation, the low and high shear wave frequencies were chosen so that there were at least one and a half wavelength encoded in the propagation direction and that a wavelength contained at least nine voxels^52^. Therefore, taking into account the size of our phantom, some pairs of frequencies between 300 and 900 Hz were considered in this work.

First, monofrequency shear waves were separately encoded with the classical MRE method. Then, shear waves resulting from the sum of the two components was encoded with the OC pulses. The TE was fixed to the minimum possible for each sequence giving an effective TE of 3.9 ms for the OC MRE and 13, 10.5, 8.8 and 7.4 ms for classical MRE with 300, 400, 600 and 900 Hz, respectively.

The waveform generator was triggered before the beginning of each TR so as to ensure a steady-state wave propagation before the encoding. Two acquisitions with inversion of the wave polarity were done for phase images subtraction and removal of static phase offsets. Acquisitions without motion were also carried out to compute the phase noise.

For the monofrequency acquisitions, four equally spaced phase offsets were acquired. For the simultaneous multifrequency encoding, the number of phase offsets was adapted to the frequency pairs so as to decrease the total acquisition duration. For instance, for the pair 300 and 900 Hz we only acquired five phase offsets equally spaced over one period of 300 Hz. This does not respect the Shannon sampling theorem, but generates a controlled temporal aliasing that enables the separation of the information of both frequencies without any additional examination time^47^. Further details of the acquisition parameters are summarized in Table 1.

**Table 1:** Acquisition parameters (frequencies f_k_ and amplitudes A_k_ of the excitation shear wave; frequency f_MEG_ and number of cycles N_G_ of the MEG; echo time, number of phase offsets, frequency resolution of the sampling $\Delta f$ and acquisition time) and results (phase encoding Δ, phase error $\sigma$ and PNRs) obtained with the different acquisition modes.

Acquisition	f_k_ (Hz)	A_k_ (V)	f_MEG_ (Hz)	N_G_	TE (ms)	#phase offsets $(\Delta f)$	Acquisition time	∆_k_ (rad)	$\sigma$(rad)	PNR_k_
Classical MRE	300	0.5	300	1	13	4 (300)	3′ 12″	1.3	0.035	21
Classical MRE	400	1	400	1	10.5	4 (400)	3′ 12″	0.46	0.037	12.4
Classical MRE	600	2	600	1	8.8	4 (600)	3′ 12″	0.58	0.030	19.4
Classical MRE	900	1.5	900	1	7.4	4 (900)	3′ 12″	0.05	0.027	1.9
OC MRE pulse A	300:900	0.5:1.5	–	–	3.9	5 (300)	4’	1.6:0.13	0.043	36.8:3
OC MRE pulse B	300:400	1:1.3	–	–	3.9	9 (100)	7′ 12″	1.2:1.3	0.067	18.4:18.7
OC MRE pulse C	300:600	1:2	–	–	3.9	5 (300)	4′	1.4:1.5	0.042	33.8:35.7
OC MRE pulse D	400:600	0.5:1.5	–	–	3.9	7 (200)	5′ 36″	2:1.8	0.048	41.4:37.3

### Image analysis

#### G′ and G″ reconstruction

Raw phase images were unwrapped with a quality guided path following phase unwrapping algorithm^53^. The 2D displacement fields, which are proportional to the unwrapped phase, were calculated. Then, a directional filter was applied in the main propagation direction (from bottom to top). A temporal Fourier transform was applied to the displacement fields along the phase offsets direction in order to obtain the different harmonics in the frequency domain. The first harmonic was kept in the monofrequency acquisitions. In the multifrequency acquisitions two harmonics were kept corresponding to the different frequency components. For example, for the pulse A, both the first and the second harmonics were kept corresponding to the 300 and 900 Hz components respectively.

Then, a spatial fourth-order 2D Butterworth filter with lower and upper thresholds of 0.25 and 2.5 cm^−1^ at 300, 400 and 600 Hz, and 1 and 3.5 cm^−1^ at 900 Hz was applied to filter and preserve the wavelengths of interest as much as possible^3,54^. The spatial filter limits were chosen based on simulations of estimated wavelength values with different cut-offs filters values (data not shown).

The shear storage modulus G′ and the loss modulus G″ at each frequency were calculated after the application of the Helmholtz equation inversion^55,56^ to the filtered displacement fields, assuming a density of 1000 kg/m^3^. Finally, a 3 × 3 median filter was applied to the elastograms.

ROIs (Region of interest) were manually drawn excluding the phantom borders and regions where the wave propagation was not sufficient (particularly at 900 Hz). The mean and standard deviation of G′ and G″ were computed inside the ROIs.

#### PNR computation

PNR, which evaluates the motion encoding efficiency, was computed and compared among the different acquisitions. It was defined as the ratio between the average value (over all pixels inside the same ROIs defined for the G′ and G″ computation) of the phase encoding in presence of mechanical excitation (∆) and the phase noise, defined as the average value of the phase encoding in the absence of motion (σ): PNR = ∆/σ. The phase encoding is defined as the difference of the maximal and minimal phase encoded in a same pixel along time. Phase encodings were calculated on phase images obtained after the application of the inverse Fourier transform to the chosen harmonics for each frequency component.

## Results

### OC pulses in the simulation framework

#### OC pulses validation

**Figure 3:**
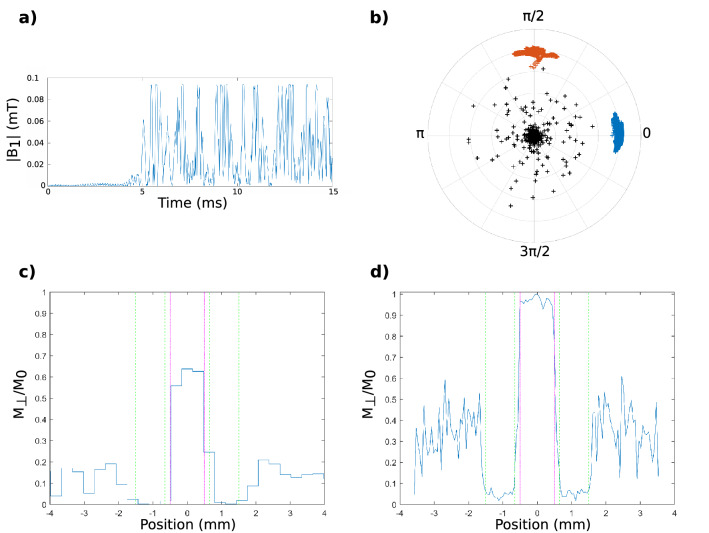
(**a**) Temporal evolution of the magnitude of the OC RF pulse A ($B_{1} = u_{x}$). (**b**) Final transverse magnetization states in the Bloch sphere after the application of the OC pulse. The null (blue) and π/2 (orange) phase isochromats are perfectly distinct. (**c**) Simulated slice profile (**d**) experimental slice profile obtained with a RF profile sequence. The same resolution parameters were used in the simulation and experimental RF profile. The pink and green dashed lines in (**c**) and (**d**) represent the theoretical controlled slice thickness and outbands, respectively.

The double role of the OC pulses of motion encoding and slice selection can be verified by simulation and experimentally. In Fig. 3, we present the characteristics of the OC RF pulse A. They are similar for the other OC pulses used in this study. The temporal evolution of the pulse magnitude is displayed in Fig. 3a. Figure 3b presents the final states of the transverse magnetization in the transverse plane of the Bloch sphere of all the isochromats considered in the optimization process. The 0 and π/2 phase isochromats are perfectly distinct. The mean phase of each isochromat group is calculated to compute the simulated encoded phase. Ideally, it should be equal to π/2. Isochromats outside the bandwidth can be identified as the group in the center of the graphic. It can be noticed however that the isochromat dispersion from the target state is more important than in the slice bandwidth. Figure 3c shows the simulated slice profile, calculated by integrating the transverse magnetization inside pixels aligned along the slice gradient direction. Figure 3d shows the experimental slice profile, obtained with a RF profile sequence. The resolution parameters were the same in the simulation of the profile and in the experimental RF profile: same number of points (128) and FOV (40 cm). In both figures, the dashed lines represent the controlled slice thickness and outbands. The experimental slice profile matches the simulated one and the obtained slice thickness corresponds to the desired one (1 mm).

#### Robustness to the T2 variability

**Figure 4:**
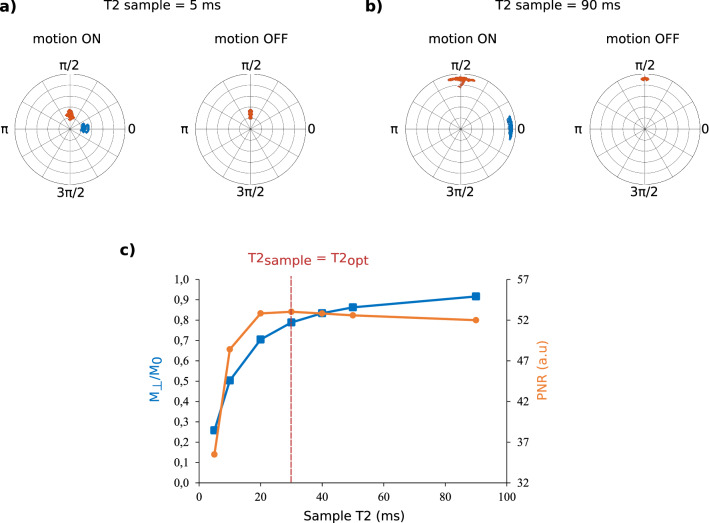
Numerical evaluation of the robustness of the OC pulse A (T2_opt_ = 30 ms) with respect to the variability of the sample T2-value. Final transverse magnetization states are presented in Bloch spheres when applying the OC pulse with and without motion for samples with T2-values of (**a**) 5 ms and (**b**) 90 ms. (**c**) Simulated transverse magnetization amplitude and PNR variation with respect to sample T2-value.

Figure 4 shows the obtained results when the numerical phantom T2 varies between 5 and 90 ms with and optimized T2 set to 30 ms. Figure 4a, b present the final states on two numerical phantoms of T2 values of 5 and 90 ms, respectively, with and without motion. We can observe that when there is no mechanical excitation, the two isochromats populations have a similar phase encoding resulting in a constant phase image. Figure 4c shows the quantified results in terms of simulated transverse magnetization in the slice and PNR. The transverse magnetization amplitude increases monotonously with T2 with a sharp increase when T2 < *T*2_*opt*_ and a slower increase for T2 values above $T2_{opt}$. PNR has maximum value when T2 = $T2_{opt}$ and decreases very slightly when T2 > $T2_{opt}$. However, an abrupt decrease is noticed when T2 < $T2_{opt}$.

### Phantom acquisitions

**Figure 5:**
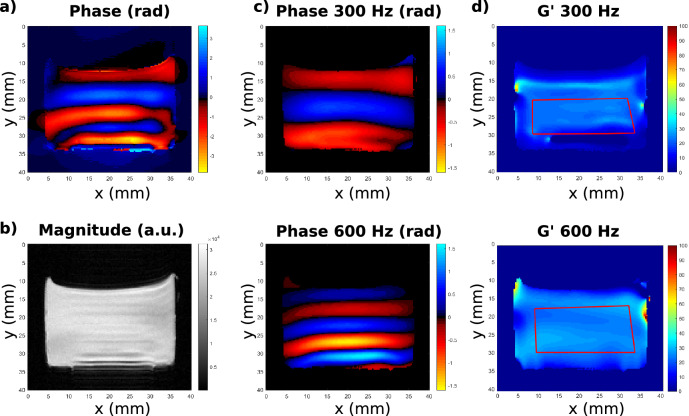
(**a**) Phase and (**b**) magnitude images of the composite excitation 300 Hz and 600 Hz acquired with the OC pulse C. (**c**) Then separated phase images (rad) of every frequency component extracted after temporal Fourier transform, choice of the corresponding harmonics and application of the inverse Fourier transform: top: 300 Hz; bottom: 600 Hz. (**d**) G′ elastograms (kPa) reconstructed for each frequency component: top: 300 Hz; bottom: 600 Hz. The ROIs used for the computation of the shear storage modulus mean and standard deviations are shown with red lines on the maps in (**c**).

Figures 5a, b show, respectively, the phase and magnitude images of the dual-frequency excitation encoded with the OC RF pulse C optimized for 300 and 600 Hz. Then, Fig. 5c shows the phase images of the two frequency components obtained after the application of the temporal Fourier transform, choice of the harmonics and application of the inverse Fourier transform. Finally, Fig. 5d shows the G′ elastograms for each excitation frequency. Even if some artifacts can be seen on the edges of the phantom, both vibrations components are correctly encoded. The maps for the remaining frequencies are quite uniform as expected since the phantom is supposed to be homogeneous.

The classical monofrequency MRE method is compared to the OC strategy in terms of phase encoding and PNR. Results are summarized in Table 1. The values of phase encoding are higher with the dual-frequency OC strategy for all the tested frequencies. This is especially favorable for the high frequency components, where the phase encoding is reduced with the classical method. However, the phase noises are also higher with the OC strategy, yielding PNR values of the same order of magnitude but usually higher than those found with the classical method.

**Figure 6:**
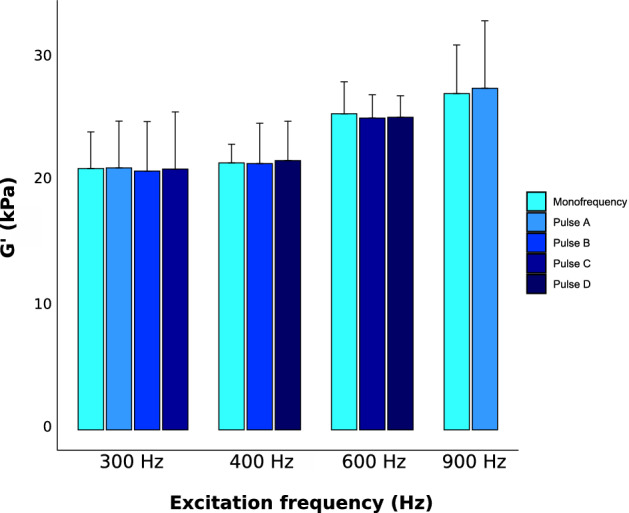
Mean values, with standard deviations displayed as error bars, of the reconstructed shear storage modulus G′ for all the different excitation frequencies obtained with the classical monofrequency and the OC multifrequency methods.

The mean values and standard deviation of G′ obtained for all the excitations frequencies with the classical and the OC RF pulses are presented in Fig. 6. Storage modulus values found in mono and (multifrequency) acquisitions were equal to 21 ± 2.9 (pulse A: 21 ± 3.8, pulse B: 20.8 ± 4, pulse C: 20.9 ± 4.6) kPa at 300 Hz; 21.4 ± 1.5 (pulse B: 21.4 ± 3.2, pulse D: 21.6 ± 3.2) kPa at 400 Hz; 25.4 ± 2.6 (pulse C: 25 ± 1.9, pulse D: 25.1 ± 1.7) kPa at 600 Hz and 27 ± 3.9 (pulse A: 27.4 ± 5.4) kPa at 900 Hz. Loss modulus values found in mono and (multifrequency) acquisitions were 5.7 ± 2.2 (pulse A: 3.8 ± 4.9, pulse B: 4 ± 3.9, pulse C: 4.6 ± 2.2) kPa at 300 Hz; 6.4 ± 1.5 (pulse B: 6.4 ± 3.8, pulse D: 6.9 ± 3.2) kPa at 400 Hz; 8 ± 2.7 (pulse C: 8.5 ± 2.9, pulse D: 8.5 ± 1.1) kPa at 600 Hz and 10.8 ± 4.1 (pulse A: 12.2 ± 4.2) kPa at 900 Hz.

## Discussion

This proof of concept proposes a novel method to carry out simultaneous multifrequency MRE. RF pulses, designed via the OC theory, are applied with a constant gradient while the shear wave propagates through the sample and have the double role of spatial selection and motion encoding. We validated on phantom experiments the ability of OC-based pulses to simultaneously encode two frequencies. We chose four different pairs of frequencies so as to cover the whole spectrum of excitation frequencies adapted to our phantom (between 300 and 900 Hz). Unlike the classical MEG MRE method, oscillating gradients are not needed after the application of the RF pulse. This implies the elimination of the constraints of the classical method due to the presence of MEGs such as high frequency oscillations and long TEs with low frequency vibrations. Hence, the OC approach is not limited in terms of frequency. However, the fast attenuation of high frequencies mechanical waves remains a physical limitation, especially for soft and deep-lying tissues. The OC MRE strategy was already demonstrated in the simpler case of single-frequency excitation^28^, hence we built upon these previous results to apply OC MRE to a dual-frequency excitation to further its benefits.

Compared to the existing simultaneous multifrequency MRE methods, with the OC approach the frequency components are not limited either to odd harmonics (harmonic wideband encoding^47^) or a narrow range of frequencies (fractional encoding^44^). Any pair of frequencies could be theoretically considered in the pulse optimization enabling exploration of the appropriate frequency range depending on the characteristics of the tissue of interest. Being able to simultaneously encode any pair or even more than two frequencies without a degraded PNR would be a valuable tool for multifrequency MRE.

The sampling method could be adapted to the driving frequencies in order to minimize the total acquisition time by using non-respecting Shannon samplings^47^ or even irregular sampling methods such as the Sequential Backward Selection (SBS) algorithm^57^. For instance, in the case of the 300–900 Hz multifrequency acquisition, a non-respecting Shannon sampling that produces a controlled frequency aliasing could be applied. It allowed to considerably reduce the number of phase offset to be acquired and thus acquisition time. Conventionally, at least 4 phase offsets equally spaced along the harmonic cycle are acquired to encode a monofrequency shear wave. This means that to separately encode two different frequencies we would need 8 phase offsets versus only 5 phase offsets with our proposed method. This method therefore enables a 38% reduction in acquisition time to encode these 2 frequencies. However, taking into account the characteristics of our phantom, not all frequency pairs could be encoded with such an undersampling method and so the acquisition time could not always be reduced that much.

According to the results, the dual-frequency OC acquisitions can produce higher PNR values compared to the classical monofrequency acquisitions, as previously found with monofrequency OC pulses^28^. A good agreement of the shear storage modulus G′ obtained through the different acquisitions is also found. Nevertheless, the loss modulus were underestimated for 300 and 900 Hz in some of the multifrequency acquisitions. This could be due to the fact that the 300 Hz wavelength is big compared to the phantom size and the 900 Hz component is rapidly attenuated. In a previous study^47^ the shear storage and loss modulus were measured by using multifrequency MRE in our phantom. It was found that G′ (300 Hz) = 20.3 ± 2.2, G′ (900 Hz) = 23.9 ± 2; G″ (300 Hz) = 6.5 ± 2.8, G″ (900 Hz) = 7.9 ± 3.8. The actual values are of the same order of magnitude but slightly higher, this could be explained because the mechanical properties of the phantom varies as it grows older (more than a year passed between the mentioned paper and the actual study).

The RF slice profiles showed in Fig. 3c, d present an imperfect null transverse magnetization beyond the controlled bandwidth and outbands rendering multi-slice acquisitions complicated. It could be possible to apply a low-pass filter with a cutoff frequency equal to the maximum value of ∆z_out_ to reduce the effect of the pulse to the magnetization trajectories lying beyond the controlled bandwith and outbands. However, post-filtering higher frequency components of the RF pulse has a detrimental effect on the transverse magnetization in the selected slice. Increasing the length of the outbands would allow the reduction of the transverse magnetization outside the slice but this would result in longer computation time.

As no oscillating gradients are required, the readout can be applied immediately after the pulse, allowing shorter echo times. Therefore, contrary to the classical method, the OC MRE approach would allow the encoding of motion in tissues with short transverse relaxation times. In this study, a spin-echo scheme was used but the application of a non-Cartesian readout is also under investigation^58^. Using a radial acquisition scheme would allow an even shorter echo time and would ultimately expand the application of multifrequency MRE to ultra-short T2 tissues, such as tendons or ligaments. Relaxation times are taken into account during the optimization process. Indeed, the OC algorithm tries to compensate the T2 relaxation to maximize the transverse magnetization at the end of the pulse. In Fig. 4c, the resulting transverse magnetization when *T*2 = *T*2_*opt*_ = 30 ms is about 80% of the maximum value even though the pulse duration is 15 ms. Such a transverse magnetization can only be obtained because during the pulse duration, the magnetization trajectory corresponds to a succession of partial flips and phase encodings. Moreover, with the OC pulses we are not limited in terms of excitation frequency. High frequencies could be applied to quantify the mechanical properties of hard tissues like cartilage^59^ to detect some diseases such as osteoarthritis^60^. But we could also encode low frequency vibrations with short TEs in soft tissues such as the liver.

One limitation of the proposed approach is that a prior knowledge of some of the acquisitions parameters is needed to compute the OC pulse. Some of them are related to the RF coil (maximal amplitude), the phantom (T1 and T2 relaxations times), the MRI scanner (gradient maximum amplitude) and the mechanical excitation (amplitudes and frequencies). An incorrect correspondence between experimental and optimization parameters can affect the efficacy of the OC pulse, especially in the case of a dual-frequency pulse where the optimization process is more complex. The effect of the deviation of the experimental parameters has been investigated with simulations for some of these parameters: from Fig. 4, we concluded that it is still possible to encode motion when the actual T2 is bigger than the optimized T2. One can also note that for T2s that are slightly smaller than the optimized value, the OC pulse is still able to fulfill correct phase encoding but transverse magnetization rapidly decreases. It was therefore demonstrated the robustness of OC pulses to the sample T2 variability, meaning that an OC pulse optimized for a minimum possible T2 could be used in a wide range of higher T2s. This would facilitate the use of the OC strategy on clinical setups since just a few pulses could be sufficient for different applications. From simulations (data not shown here), we found that the phase is still encoded when the experimental amplitudes are smaller than the amplitudes used for the optimization. This means that OC pulses can encode the attenuation along the sample. However, having experimental amplitudes bigger than the optimization one could prevent the encoding of motion. Therefore, the amplitude of vibrations has to be fixed carefully for the optimization process and in such a way that the highest experimental amplitude is below the optimization one.

For the moment, we validated the use of OC RF pulses to perform multifrequency MRE in preclinical experiments where higher gradients are available. However, OC RF pulses could also be adapted to clinical requirements. We optimized some RF pulses with gradient amplitudes of the order of magnitude of clinical gradient systems (< 40 mT/m). Such pulses were tested in our 7T MRI scanner and demonstrated their ability to encode a dual-frequency shear wave. This confirmed that the application of OC RF pulses to encode motion is not limited by the strength of the gradient system. Furthermore, as any pair of frequencies can be considered in the optimization, it could be easily adapted to the clinical elastography frequency range. The physical limitation of higher attenuations at high frequencies could be overcome with a mechanical actuator using centrifugal force. Such an actuator was demonstrated to provide sufficiently large wave actuation at high frequencies (from 50 to 80 Hz at 3 T)^61^. Vibration frequencies intrinsic to the MRI and the sequence should be evaluated before the transfer of the OC strategy on a clinical MRI scanner to adapt the vibration frequencies and the sampling strategy. Finally, the actual limitation of the OC pulses to only encode oscillatory motion vibrating in the same direction as the constant gradient is under investigation to expand the strategy to 3D motion encoding.

In conclusion, this article is the proof of concept of an innovative simultaneous multifrequency MRE method in a preclinical context. OC pulses have proven to be a valuable tool to encode a dual-frequency motion without the need of oscillating motion encoding gradients. Even if this novel approach presents some limitations, it is still advantageous for multifrequency MRE thanks to the resulting shorter acquisition time and achieved short echo times. We have shown that with OC pulses, a dual-frequency motion can be encoded and the viscoelastic parameters quantified, with comparable results to the classical MRE method. Future work will focus on expanding the motion encoding to more than one direction and applying the OC strategy with radial readouts to perform MRE in very short T2 tissues. Preclinical in vivo studies will be conducted on small animals in order to evaluate the encoding efficiency of the OC simultaneous multifrequency MRE approach.

## Conclusion

This article presents an alternative strategy to the current simultaneous multifrequency MRE methods: applying a designed OC pulse with a constant gradient, it is possible to encode a shear wave composed of low and high frequency components without a degradation of the encoded phase. This strategy can be combined with an undersampling method in order to reduce the total acquisition time. The multifrequency OC approach could allow the mechanical characterization of short T2 tissues as it is compatible with short echo times and it could also be useful to explore the dispersion of viscoelastic parameters of tissues on a large frequency range. Further work is envisaged to investigate the preclinical applications of the presented method.

## Data Availability

The datasets generated during and/or analyzed during the current study are available from the corresponding author on reasonable request.
